# Biodistribution, migration and homing of systemically applied mesenchymal stem/stromal cells

**DOI:** 10.1186/s13287-015-0271-2

**Published:** 2016-01-11

**Authors:** Johannes Leibacher, Reinhard Henschler

**Affiliations:** Institute of Transfusion Medicine and Immune Hematology, German Red Cross Blood Donor Service, University of Frankfurt, Frankfurt, Germany; Blood Donor Center Zürich, Swiss Red Cross, Zürich, Switzerland

## Abstract

Mesenchymal stem/stromal cells (MSCs) are increasingly used as an intravenously applied cellular therapeutic. They were found to be potent in situations such as tissue repair or severe inflammation. Still, data are lacking with regard to the biodistribution of MSCs, their cellular or molecular target structures, and the mechanisms by which MSCs reach these targets. This review discusses current hypotheses for how MSCs can reach tissue sites. Both preclinical and clinical studies using MSCs applied intravenously or intra-arterially are discussed in the context of our current understanding of how MSCs might work in physiological and pathological situations.

## Background

In the 1970s, Friedenstein and colleagues [1] first reported that locally applied culture-expanded populations of bone marrow stroma-derived fibroblastic cells remained at their injection sites under the kidney capsule, where an ectopic hematopoiesis was initiated. Later, Arnold Caplan’s group described mesenchymal stem/stromal cells (MSCs) as multipotent mesenchymal cell populations which can differentiate into several tissue types, and demonstrated roles for MSCs in the regeneration of bone, cartilage or ligaments in animal and clinical studies [2–4]. In these studies, however, transplanted cells were followed, if at all, at the site of transplantation, and biodistribution was not an issue.

By the year 2000, clinicians had become increasingly interested in intravenously applied MSCs. Pivotal studies by the group of Horwitz in children with osteogenesis imperfecta, an inherited enzyme deficiency of collagen synthesis by mesenchymal cells in bone, opened the field for intravenous use of MSCs. This concept started from the observation that bone marrow transplantation can provide stromal cells able to synthesize intact collagen type I, replacing deficient patient cell function and ameliorating disease symptoms [5]. Therefore, the authors concluded that transplantation of isolated healthy allogeneic MSCs might cure the disease. This implies homing of transplanted MSCs to sites in bone marrow and/or bone. Efficacy was noted in all six infants treated [5]. Children who received transplants showed improved growth rates and started to synthesize intact bone. Engraftment of donor-type MSC-derived osteoblasts was shown using bone specimens and microsatellite DNA marker analysis. In a second study [6], these authors showed that autologous, enzyme-deficient MSCs transduced with a copy of the intact gene resulted in normal collagen production in bone cavities. Moreover, children who received transplants approached growth curves similar to the children transplanted with allogeneic complete bone marrow [6]. This pioneering work provided the basis for the successful application of MSCs using the intravenous route in other clinical entities.

## Establishment of methods to track intravenously administered MSCs

After 2000, the therapeutic use of MSCs by intravenous administration was explored by a number of studies in animals and also humans. These studies used various ways to label culture-expanded MSCs, and to track them in different tissues over time. The tissue source of the MSCs was in most cases not decisive, and cells from various tissue sources were explored. The labeling methodologies used included radioactive labeling of MSCs, labeling with fluorescent vital dyes, contrast agents, transduction with reporter genes, or the use of donor cell-specific DNA markers such as microsatellites [7–11] (reviewed in [12]). The labeling methodologies were, in part, designed to detect only short-term homing of MSCs. In addition, they do not enable the determination of whether detected cells are still alive. These studies were mainly conducted in rodents and nonhuman primates and mostly in non-injury situations. The main common results of these studies were that: MSCs distribute to a variety of tissues after intravenous (i.v.) injection; MSCs are detectable at low or very low frequencies in tissues after transplantation; and signals from the injected cells were found early after administration of the MSCs at the highest frequencies in the lungs, followed by liver and spleen.

The observed biodistribution patterns were confirmed by studies in humans. In patients with mammary carcinoma, Koç et al. [13] demonstrated that i.v. MSCs were well-tolerated in patients at a dose of one million MSCs/kg body weight; however, the cells were trackable in blood only. The data were confirmed in patients with liver cirrhosis using ^111^In-oxine labeled MSCs, which were found to first accumulate in the lungs followed by continuous increases in liver and spleen up to day 10 after administration [14]. The proportion of accumulation in lung decreased from about 35 % early after transplantation to 2 % or less by day 10, whereas spleen had the highest signals by day 10 after transplant. These results confirm a similar overt biodistribution of MSCs in lung, liver and spleen in humans to that observed in animal models.

## Expression of cell adhesion molecules by MSCs as a basis for their interaction with endothelial cells and tissue-directed extravasation

In theory, the main prerequisite for the interaction of transplanted MSCs with endothelial cells are adhesion molecules present on the cell surface of MSCs, and expression of appropriate adhesion counter-receptors on endothelial cells. MSCs (most investigations were performed in human MSCs (hMSCs)) have shown deficits in receptor binding to selectins and/or their ligands. They lack expression of L-selectin, and their E-selectin ligand (CD44) is not functional [15]. MSCs can bind to P-selectin through a fucosylated ligand, which nevertheless is not P-selectin glycoprotein ligand (PSGL)-1 [16]. Thankamony and Sackstein [17] have, however, defined an enzymatic fucosylation procedure which causes the CD44 epitope on MSCs to strongly bind to endothelial E-selectin, resulting in effective rolling of MSCs on endothelial cells and, moreover, extravasation into bone marrow sites. Of the integrins, alpha4beta1 (VLA-4) and alpha5beta1 (VLA-5) have been found to be expressed by MSCs, whereas the beta2 integrins alphaLbeta2 (LFA-1) and alphaMbeta2 (Mac1) could not be detected [15, 16, 18–20] (reviewed in [12, 21]). Interestingly, several chemokine receptors have been found to be expressed on MSCs, including CXCR4, which has been described as a major mediator of the homing and mobilization of hematopoietic cell types [12, 19, 20]. In summary, these findings indicate that MSCs have a deficit with regard to the expressing and/or employing adhesion receptors for coordinated extravasation and tissue-specific homing, as do leukocyte populations.

## Emergence of common themes in exploring the biodistribution of MSCs

Subsequent to the first reports on the homing and migration of transplanted MSCs to tissues, additional questions about MSC biodistribution have been addressed, including quantification of MSCs, their preferential homing to several target sites, and the involvement of cues, such as regeneration or inflammation, and the size of MSCs in determining their biodistribution (Table 1).

**Table 1:** Common themes in MSC biodistribution research

Theme	Targeted tissues (possible mechanism)	References
Increased homing after intra-arterial delivery compared with intravenous delivery?	Kidney	[33, 34]
	Joints	[32]
	Stroke	[30]
	Other (many) tissues	[31]
Side effects of intra-arterial versus intravenous delivery?	Incorporation into vessel wall	[23, 35]
	Obstruction of microvessels	[38]
	Vascular occlusion	[39]
Targeting of vessel wall and vessel-associated tissues?	Lungs, lymph nodes, intestine	[47]
Targeting of tissues for regeneration	Myocardium	[18, 48–55]
	Beta1 integrins	[48, 49]
	CCL2, monocytes	[52]
	Kidney	[33, 56–63]
	Gut and liver	[64–67]
	Skin	[44, 68–71]
	CCL21	[44]
	JAM-A	[68]
	Brain	[72–75]
	P/E selectin (CD44)	[73]
	CXCR4/flk-1/EPO-R	[74]
Homing to bone marrow	Bone marrow	[76–81]
	HCELL/E-selectin	[15]
	Subendothelial localization	[79]
Biodistribution to the immune system?	Macrophages	[37, 41, 42]
	Dendritic cells	[38]
	T cells	[39]
	Unknown target cells	
	Idoleamine desoxygenase	[43]
	Prostaglandin E2	[37, 41]
Elimination mechanisms?	Antibody formation	[6]
	Phagocytes	[102]
Influence of radiation on homing?	Increased in brain, heart, bone marrow, and muscles	[43, 82]
Homing in malignancies?	Tumor	[83–85, 87–92]
	Mediated by CCL25	[88]
	Mediated by sodium iodide symporter under the control of RANTES/CCL-5 promoter	[87]
	Homed MSCs form tumor-associated fibroblasts	[90]
Formation of microvesicles	Microvesicles may contribute to/be part of MSC biodistribution	[14, 63, 93–97]
	Mediated by horizontal transfer of microRNAs	[96]

*MSC* Mesenchymal stromal/stem cell

In many of the earlier studies, the target sites as well as the molecular mechanisms governing the interactions of MSCs with the local environment after transplantation (e.g., endothelial cells, target tissue), such as adhesion molecules or signaling mechanisms, were either not addressed or were analyzed only to a minor degree. Moreover, MSCs were often evaluated by microscopy, a method relatively prone to artifacts. Many studies also did not quantify the numbers of MSCs in target or other tissues. Likewise, only few studies reported on the size of the identified MSCs. Despite this lack of information, other themes have emerged, especially research on cues that may regulate the biodistribution of systemically applied MSCs; these include first pass tissues, specifically the lungs, inflammation, irradiation, sites of hypoxia or repair, and cancer (Table 1). As a result, concepts have been raised which imply an ability of MSCs to migrate to specific sites—e.g., MSCs as an “injury drugstore” for several acute clinical situations [21, 22].

## First-line accumulation of intravenously administered MSCs in the lungs

The first hurdle for intravenously transplanted MSCs is the lung capillary bed. After culture expansion, MSCs are relatively large cells with an estimated average size of around 30 μm in suspension (ranging from 16–53 μm) [23]. Their size may also vary depending on the osmolarity of the culture media, passage number, and/or cell density during seeding as well as general culture conditions (two-dimensional versus three-dimensional culture). In comparison with MSCs, hematopoietic stem cells have a much smaller diameter, ranging from 4–12 μm depending on the subfraction analyzed [24, 25]. Therefore, obstructive events during lung passage are expected after intravenous administration of MSCs. Lee et al. [26] presented a kinetic study of MSCs accumulating in murine lungs in which up to 80 % of injected cells were found in the lungs within a few minutes after injection. Moreover, formation of emboli in lung vessels was noted. The MSC signal (an *Alu* sequence DNA marker) fell exponentially, with a half-life of about 24 h and practically complete disappearance after 4 days [26]. Barbash and colleagues [10] confirmed the detection of the overall MSC load in the lungs using ^99m^Tc-labeled MSCs in a rat model with induced myocardial infarction. Murine MSCs also showed deleterious effects in mice, including post-injection lethality, which was not the case after administration of hMSCs [27]. Interaction of human or murine MSCs with lung endothelial cells was dependent on the suspension medium in which the transplanted cells were administered [27]. Adhesion of the MSCs to endothelial cells was found to involve the integrin ligand vascular cell adhesion molecule (VCAM)-1. When comparing MSCs with mononuclear cells from bone marrow, neural stem cells and multipotent adult progenitor cells, Fischer et al. [28] found that MSCs showed the highest interaction with lung endothelia, which could be inhibited by pretreatment with anti-CD49d antibody. In a study by Kerkelä et al. [29], adhesion of MSCs to lung tissue (probably endothelial cells) was dependent on the enzyme treatment used during harvesting of confluent MSCs in culture before transplantation; after treatment with pronase, MSCs more readily cleared the lungs and could be found in other tissues compared with trypsinization treatment. Taken together, these data indicate an active role of the adhesion molecules VLA-4/VCAM-1 on MSCs/endothelial cells during interaction of MSCs with lung tissue. It remains to be clarified, however, whether this is a passive or active process. Also, relatively little is known about possible adhesion molecules other than VLA-4/VCAM-1 which may be operative in the interaction of MSCs with endothelial cell surfaces in the lung. This includes the fucosylation of CD44 to HCELL, a highly active E-selectin ligand on MSCs, which is relevant in bone marrow endothelia but seemingly did not affect lung interactions [15].

In summary, presently there is strong evidence that accumulation of MSCs in the lungs is a key determining factor for their biodistribution. The major adhesion molecule involved seems to be VLA-4/VCAM1. Still, it is not clear to what degree the findings in animal studies are quantitatively transferable to humans (Table 1).

## Biodistribution of MSCs after intra-arterial versus intravenous administration

Studies comparing intra-arterial and intravenous application of MSCs have demonstrated a major association between intravenous application and retention of MSCs in the lungs, and their increased accumulation in therapeutic target tissues after intra-arterial injection. Walczak et al. [30] in a rat transient ischemia stroke model applied two independent detection methods (magnetic resonance imaging and Doppler flowmetry). They demonstrated that higher cerebral engraftment rates are associated with impeded cerebral blood flow, and that intra-arterial delivery may be advantageous in ischemic stroke to deliver MSCs to the site of injury. Mäkelä et al. [31] compared intra-arterial and intravenous administration of MSCs labeled with ^99m^Tc, and also found that the intra-arterial transplantation route has a positive impact on the biodistribution of bone marrow-derived MSCs (BM-MSCs) to peripheral tissues. They found that intra-arterial transplantation decreased the deposition of BM-MSCs in the lungs and increased uptake in other organs, especially in the liver. In a study looking at human adipose tissue-derived MSCs in SCID mice, Toupet et al. [32] showed that 15 % of intra-arterially injected MSCs accumulate in inflamed joints during the first month, and 1.5 % over a longer term of >6 months, also favoring intra-arterial over intravenous application for, in their case, anti-inflammatory MSCs. Therapeutic effects of MSCs in kidney have been generally achieved after intra-arterial delivery [33, 34]. Although more studies will be needed, these data suggest that the intra-arterial route of administration is effective in avoiding pulmonary entrapment of BM-MSCs, and may thus improve the biodistribution and bioavailability of transplanted MSCs in clinically relevant tissues for, e.g., tissue repair.

## Interactions of MSCs with the blood vessel wall: integration into the vessel wall or transmigration?

As described above, the majority of intravenously injected MSCs are generally detected in the lungs, and in no other tissue at comparable numbers even at later time points. Some groups asked whether MSCs may directly target vessels or perivascular tissue and investigated the fate of MSCs in and around blood vessels. These studies followed the cells using intravital microscopy and histologic examination in different tissues after intra-arterial [23, 30, 35] administration. In the cremaster muscle intravital microscopy model, Furlani et al. [23] observed that the microcirculation was disturbed, with some MSCs obstructing small vessels. In addition, pulmonary emboli were found. Toma et al. [35] also observed occlusion of microvessels and entrapment of the injected MSCs. Moreover, they observed stable integration of some transplanted cells into the vessel wall. Cui et al. [36] reported a risk of vascular occlusion in their rat stroke infarction model after intra-arterial injection, pointing to the fact that local intravasal entrapment of MSCs may frequently occur, and MSCs may obstruct the microcirculation. Currently, however, we lack conclusive data that MSCs that are entrapped in capillaries and/or are incorporated into the vessel wall or adjacent to endothelial cells would relocate (i.e., “home”) to their main tissue of origin, pericytes.

## Transplanted MSCs interact with cells of the immune system

Transplanted MSCs have been shown to rapidly interact with immune cell types, which are—at least in part—present also in the bloodstream. In a lung sepsis model, Nemeth et al. [37] observed that MSCs co-localize with lung-resident macrophage cells and induce them to produce anti-inflammatory interleukin (IL)-10 via release of prostaglandin E by MSCs as part of their therapeutic effect. Chiesa et al. [38] showed that interstitial dendritic cells (DCs) decrease their physiological migration from skin to lymph nodes rapidly after intravenous administration of MSCs. They describe that MSCs inhibit Toll-like receptor (TLR)-4-induced activation of DCs, which results in the inhibition of cytokine secretion by DCs, downregulation of adhesion molecules involved in the migration of DCs to the lymph nodes, suppression of DC antigen presentation to CD4+ T cells, and cross-presentation to CD8+ T cells. Akiyama et al. [39] demonstrated that both human and murine MSCs can induce immune suppression by attracting and killing autoreactive T cells through FasL, thereby stimulating transforming growth factor beta production by macrophages and generation of regulatory T cells. The interaction has been shown to involve the secretion of MCP-1 by MSCs. The dying T cells in turn activate macrophages to produce transforming growth factor beta, thus stimulating regulatory T cells and promoting immune tolerance. Possibly, the secretion of anti-inflammatory protein TSG-6 by activated MSCs, which has been described in a zymosan-induced mouse peritonitis model, involves an interaction via TLR2/reduction of NF-κB signaling in resident macrophages [40].

Another type of potential interaction between MSCs and immune cells is suggested by data from Kim et al. [41], who used an in vitro system showing that murine MSCs inhibit functionality of DCs through TLR-4-mediated signals in co-culture with monocytes. During this study, hMSCs revealed a unique immunophenotype of alternatively activated human monocytes which are CD206-high, IL-10-high, IL-6-high, IL12-low, and tumor necrosis factor (TNF)-alpha-low [42]. The immune suppressive effects of MSCs have been shown to depend on induction of indoleamine 2,3-dioxygenase [43], or production of prostaglandin E2 as a main effector to dampen inflammation [37, 44]. These data indirectly support the hypothesis that MSCs interact directly with monocytic and/or antigen-presenting cells in vivo.

The successful therapeutic use of MSCs in patients with severe immune dysregulations, such as graft-versus-host disease after allogeneic hematopoietic stem cell transplantation, has attracted high interest by hematologists (reviewed in [45]). The studies were based on a number of in vitro findings that MSCs can either interact with or affect the function of various types of immune effector cells such as antigen-presenting cells, B or T lymphocytes, or natural killer (NK) cells (reviewed in [46]). In all these studies, identification of MSCs at target sites has been cumbersome, and often no transplanted MSCs were identified. von Bahr et al. [47] reported that MSC donor DNA was detectable at low levels in 8 out of 18 patients in vessel-associated tissues in the patients, including lungs, lymph nodes, and intestine. Detection of MSC donor DNA was negatively correlated with time from infusion to sample collection.

Together, these studies strongly indicate the existence of interactions between transplanted MSCs and cells of the immune system. This way, MSCs also biodistribute to the immune system through contact with different types of leukocytes in the circulation or various tissues such as skin, spleen, and lymph nodes.

## Potential mechanisms of elimination of MSCs from the circulation

A relevant aspect of the interaction between transplanted MSCs and immune system cells, in both animal models and humans, is the induction of xenogeneic and allogeneic immune responses, resulting in antibody formation or T-cell responses against the transplanted MSCs. Induction of antibody formation explains the failure to identify transplanted MSCs in patients upon repeated administration of allogeneic MSCs that had been cultured in fetal bovine serum-containing media [6]. Anti-fetal calf serum antibody formation has been demonstrated in patients that did not respond to repeated MSC applications [6]. Elimination of xenogeneic MSCs in some of the animal models studied may occur in ways analogous to those in the allogeneic situation.

Despite the fact that several target tissues of MSCs have been established, there are few data as to the place to which systemically applied MSCs will finally migrate, or where they end up before or when they are eliminated. The fact that the transplanted MSCs are often not detectable at all, or only a small fraction of them is traced, underscores the potential relevance of the lung as a “first pass” tissue, and may indicate an involvement of lung trapping in elimination of MSCs. On the other hand, the fact that MSCs are barely or not at all detectable in patients after transplantation demonstrates that systemic pathways to eliminate transplanted MSCs may be operating in humans, leading to barely detectable long-term engraftment.

## Tissue repair situations which provide cues to attract transplanted MSCs

The interactions of MSCs with different types of immune cells point to their ability to respond to signals from the immune system. Since aspects of tissue repair have been associated with (adaptive) immune responses, it is likely that inflammatory and tissue repair signals influence MSC responses in vivo, including their biodistribution.

### Myocardial infarction

The VLA-4/VCAM receptor axis has been shown to be involved in MSC migration in myocardial infarction. Pre-treatment of MSCs with TNF-1alpha stimulated migration of MSCs through heart endothelia mediated through VCAM-1, indicating that beta1 integrins are actively involved in this process [48]. Confirming this hypothesis, Ip et al. [49] demonstrated in a murine model that alpha4 integrin is required for migration of MSCs to myocardium, whereas the chemokine receptor CXCR4 was dispensable for the entry of transplanted cells into ischemic tissue.

Intravenously administered MSCs have been observed to, at least transiently, accumulate in areas of myocardial ischemia [18, 50, 51]. To this end, Belema-Bedada et al. [52] employed a transgenic mouse model expressing the monokine CC-chemokine ligand (CCL)2 under a cardiac specific promotor, increasing CCL2 expression in heart muscle. These authors observed that i.v. MSCs accumulate rapidly and selectively in the heart. They showed that the migration of the MSCs to heart is preceded by monocyte emigration to the myocardium, and involves G-protein-coupled receptors, pointing also towards the involvement of chemokine signals. Kraitchman et al. [11] confirmed the accumulation of i.v. MSCs into myocardial infarction areas using a radioimaging tracer and single-photon emission computed tomography in a dog model. Wang et al. [53] traced MSCs at later stages after infarction, and saw markers of newly regenerated cardiomyocytes. It is also not clear whether MSCs steadily incorporate into cardiac tissue. Other studies have failed to detect any homed MSCs in cardiac tissue over the long term (e.g., [54]). Jasmin et al. [55] injected MSCs i.v. after nanoparticle labeling in a model of heart inflammation caused by the Chagas disease parasite *Trypanosoma cruzi*. They observed that although most MSCs migrated to the lungs, liver and spleen, a few cells homed to the inflamed heart. In conclusion, some mechanisms seem to recruit, mostly transiently, some MSCs to inflamed or ischemic heart, including VLA-4/VCAM-1 and the CCL2 and possibly other chemokine receptor signals.

### Kidney damage

Despite the wide range of beneficial effects seen with the therapeutic use of MSCs in animal models, only a few clinical trials have tested the efficacy of MSCs for renal diseases. Reinders and colleagues [56] used intravenous injection of 1 × 10^6^ autologous BM-MSCs/kg in six kidney allograft recipients to dampen rejection of the graft and/or decrease interstitial fibrosis and tubular atrophy. Likewise, Tan et al. investigated autologous BM-MSCs (1–2 × 10^6^/kg) at kidney reperfusion and 2 weeks after application; the incidence of acute rejection decreased and renal function at 1 year improved compared with anti-IL-2 receptor antibody induction therapy [57]. In a clinical phase I safety trial, five patients aged >65 years with underlying renal disease and multiple comorbidities were infused with allogenic MSCs during coronary artery bypass or cardiac surgery. Although the follow-up period was short and one of the patients died, none of the patients required dialysis, supporting the beneficial influence of MSCs on repair of kidney damage [58, 59].

In animal studies, MSCs were also associated with repair of the permeability barrier of the glomerulus in an Alport disease model [60] and improved kidney function in an experimental sepsis mouse model through reprogramming of macrophages via release of prostaglandin E2 [37]. Morigi and colleagues [61, 62] have shown that treatment with murine BM-MSCs (2 × 10^5^ per mouse) in an acute renal failure mouse model induced by cisplatin (a nephrotoxic anti-cancer drug) protected the animals from renal function impairment and tubular injury. Intriguingly, the effects of MSCs in stimulating proliferation and inhibiting apoptosis of tubular epithelial cells in a glycerol-induced acute kidney injury SCID mouse model could also be achieved by using microvesicles derived from hMSCs [63]. In addition to these human studies, several studies demonstrate that MSCs localize within injured kidneys when injected in mice with acute kidney injury (e.g., [34, 63]; reviewed in [58]). The presence of MSCs at later stages of kidney injury or regeneration has not been studied, but the therapeutic benefits have been measured, and intra-arterial injection of MSCs seems to be more favorable [33, 34, 61].

### Liver damage

Gholamrezanezhad et al. [14] studied i.v. infused ^111^In-oxine-labeled MSCs in patients with liver cirrhosis. The radioactivity was first observed to accumulate in the lungs. During the following hours to days, the radioactivity gradually increased in the liver and spleen, with spleen uptake exceeding that in the liver in all patients. In the liver and spleen, radioactivity increased by day 10 post-infusion, whereas residual activity in the lungs decreased approximately tenfold. In contrast, Briquet et al. [64] saw no recruitment of hMSCs to liver damaged by CCl4 intoxication in immune-deficient mice. A study by Zhang et al. [65] indicates that corticosteroids and the SDF-1/CXCR4 axis are involved in MSC migration in a carbon tetrachloride-induced liver fibrosis model. Another liver regeneration model in mice indicated that MSC homing to liver was associated with regeneration, but the mechanisms for this were not investigated [66]. In summary, although many of the published studies have not addressed aspects of MSC biodistribution, there is some evidence for biodistribution to injured or diseased livers, but the underlying mechanisms are mostly unclear.

### Gut and skin

Only a few studies have analyzed MSC accumulation in epithelial tissues so far. Inflammatory bowel disease models have addressed homing of i.v. MSCs. Parekaddan et al. [67] demonstrated the presence of MSC-derived signals not only in lungs and spleen but also in the gut of affected animals. Sasaki et al. [44] assessed whether homed MSCs can differentiate into skin cells, including keratinocytes, and whether they could contribute to wound repair. They i.v. injected green fluorescence protein (GFP) transgenic MSCs and identified GFP-positive cells associated with specific markers for keratinocytes, endothelial cells, and pericytes. They attribute the extravasation to inflamed areas to the presence of the chemokine CCL21 in vessels in the inflamed tissue. Still, numbers of detected MSCs in the wounded skin areas were low. MSCs have been found in wound tissues several days after transplantation in animal models [68–71] but their engraftment efficiency ranged from <0.01 % when MSCs were intravenously injected to 3.5 % in a study where MSCs were locally applied. This points to a minor role of i.v. injected MSCs in skin repair. One study reported that, after intravenous injection of GFP transgenic MSCs, keratinocytes, endothelial cells, pericytes and macrophages within the healed wound were all found to be GFP-positive. The authors concluded that they might be derived from donor MSCs [71].

### Brain

Some studies have investigated whether transplanted MSCs migrate into inflamed brain tissue. In murine stroke models, MSCs migrated into ischemic areas after intravenous delivery [72, 73]. The latter study mentions that the MSCs are recruited to these sites via endothelial expressed P- and E-selectin, and that CD44 is present on the MSCs. In their rat brain ischemia model, Wei et al. [74] found that i.v. MSCs localize to ischemic zones and deliver neurotrophic factors. This occurs at an increased rate when MSCs have been exposed to hypoxia before transplantation. The extravasation efficiency of the MSCs correlated with increased expression of CXCR4, flk-1 and the erythropoietin receptors, and downregulation of pro-inflammmatory regulators in the homing MSCs. The activity of microglia formation was suppressed in animals after MSC therapy, and NeuN-positive and Glut1-positive cells were increased. Constantin et al. [75] used intravital microscopy in a murine experimental autoimmune encephalitis model. They found, using bioluminescence, accumulation of a subset of transplanted MSCs in inflamed brain venules in inflammatory foci of experimental autoimmune encephalomyelitis 16 and 30 days after transplantation, and showed a role for alpha4 integrin in the migration process of MSCs into brain tissue. Although absolute numbers of transplanted MSCs were not determined and may be low, the results indicate that active inflammation may switch the homing behavior of transplanted MSCs from unspecific entrapment to specific recruitment.

Together, these data indicate that MSCs can migrate into ischemic and proinflammatory regions in certain disease models. Mostly short- (within the first 3 days) and mid-term (3 days to 3 months) homing has been reported, whereas long-term persistence (>3 months) of MSCs is rarely detected. Due to the technologies used to detect transplanted cells, there is only limited evidence to indicate whether the MSCs home as intact cells into their target environments. The data are in favor of transient homing and locally acting MSCs in the investigated pathologies.

## Homing of transplanted MSCs to bone marrow

Several decades of clinical and experimental work in the field of bone marrow transplantation have shown that donor type MSCs will generally not engraft in allogeneic hosts, including the precursor cell type for MSCs, fibroblast colony-forming units [76–78]. Rombouts and Ploemacher [79] demonstrated that prolonged time in culture induces a defect in MSCs that affects their engraftment into bone marrow in a classic bone marrow transplantation situation. In contrast, as reported above, Horwitz and colleagues [5, 6] demonstrated that MSCs engraft into bone marrow of children with osteogenesis imperfecta. Possibly, engraftment of MSCs therefore requires a “niche” which is not free in normal bone marrow transplant recipients, but is created in a deficiency state such as the collagen synthase defect found in osteogenesis imperfecta. Follenzi et al. [80] recently demonstrated that mice suffering from hemophilia A, when transplanted with normal healthy total bone marrow cells, show engraftment not only of hematopoietic cells but also of subendothelial MSC-like cells. Interestingly, these MSCs had not been cultured before transplantation. Functional MSCs may, therefore, engraft, at least in the case of certain deficiencies in the transplanted hosts. Interestingly, the group of Horwitz more recently showed that non-plastic-adherent bone marrow cells engraft in a murine model and give rise to osteoprogenitors, which are more potent osteoprogenitors than “classic” plastic-adherent MSCs in mice [81]. This underscores the possibility that the culture period induces the engraftment defect, and that, in addition, cells other than “classic” MSCs can mediate stromal engraftment. On the other hand, “classic” plastic-adherent MSCs have been shown to remain as a source of hematopoietic environment when transplanted into tissues other than bone marrow [1]. In contrast to these findings, the model by Sackstein et al. [15], where an active E-selectin ligand was engineered on the surface of plastic-adherent MSCs, resulted in efficient homing to bone marrow, indicating the possibility of BM-MSCs (or MSCs from other tissue sources) distributing to bone marrow.

## Influence of irradiation on migration and biodistribution of MSCs

In a murine study, Francois et al. [43] showed that both total body irradiation and local irradiation (e.g., selective irradiation of abdomen or legs) affected the distribution of i.v. infused hMSCs in NOD/SCID mice compared with untreated animals. Intravenously infused hMSCs were found only in minimal amounts exclusively in the lung, bone marrow, and muscles in non-irradiated control animals. Mice after total body irradiation had increased absolute numbers of hMSCs in brain, heart, bone marrow, and muscles. Moreover, selective radiation of limbs or the abdomen yielded increased engraftment of hMSCs in the exposed skin or muscles than with total body irradiation alone. hMSC engraftment outside the locally irradiated regions was also increased, pointing to both local and systemic effects of irradiation on MSC engraftment. The study did not investigate long-term engraftment. Sémont et al. [82] looked at the engraftment and efficacy of transplanted MSCs in an immunodeficient mouse model of radiation-induced gastrointestinal tract failure. They demonstrated accelerated recovery in the group receiving hMSCs, with decreased apoptosis of epithelial cells and increased proliferation within the small intestinal mucosa. Yet, transplanted MSCs were not detected at significant amounts.

## A special case: migration and engraftment of MSCs into tumors

Tumor-associated fibroblasts have been described as a form of MSCs, which are recruited from the MSC pool and are an integral part of the microenvironment of many different solid tumors [83, 84]. Tumor tissue therefore also represents a target for the homing of i.v. injected MSCs. In experimental studies, both beneficial and adverse effects have been reported. Beckermann et al. [85] verified the migration of i.v. MSCs into areas close to the vessel wall in human pancreatic tumors in immunodeficient mice. Alieva et al. [86] followed locally implanted adipose tissue-derived MSCs with a genetic modification induced by lentiviral transduction and traced them by bioluminescence in a glioblastoma model. After incorporation of the transplanted MSCs, administration of gancyclovir activates the thymidine kinase transgene, resulting in death and elimination of the transplanted MSCs and tumor regression. A PECAM-Promotor-driven second transgene as reporter construct served to indicate that the transplanted MSCs can acquire endothelial-like characteristics. Similarly, Knoop et al. [87] used i.v. MSCs expressing sodium iodide symporter under the control of the RANTES/CCL-5 promoter; when loaded with ^131^I compound these conferred significant anti-tumor effects.

Xu et al. [88], in a myeloma model, showed that MSCs are chemoattracted by the chemokine CCL25, thus supporting myeloma growth. In a Ewing sarcoma nude mouse model, i.v. injected MSCs expressing IL-12 were effective in treating the sarcomas [89]. Interestingly, the transplanted MSCs themselves were not identified, while the secreted IL-12 was. Kidd et al. [90] showed that tumor-associated fibroblasts originating from transplanted MSCs in syngeneic ovarian and breast cancers are recruited from the bone marrow, whereas the bulk of the vascular and fibrovascular stromal cells (pericytes, α-smooth muscle actin-positive myofibroblasts, and endothelial cells) were recruited from adipose tissue. These data indicate a process whereby, once bone marrow homing of transplanted MSCs is established, these MSCs may be (genetically) directed along pre-established pathways of endogenous MSCs that circulate from bone marrow to the tumor. Further work by Grisendi et al. [91] demonstrated that the process of MSC incorporation into tumors implies the formation of epithelial–mesenchymal or endothelial–mesenchymal transitions, and requires the formation of fibroblasts derived from mesenchymal progenitors.

MSCs were also found to enhance angiogenesis, as shown in models of B16 melanoma cells and Lewis lung carcinoma [92]. Co-injection of tumor cells and MSCs led to increased tumor size compared with injection of tumor cells alone. Tumor vessel areas were greater in tumors after co-injection of tumor cells with MSCs than in tumors induced by injection of cancer cells alone. Co-injected MSCs localized close to vascular walls, and also expressed the endothelial marker CD31/PECAM-1.

In conclusion, MSCs show a clear tumor tropism. Many data indicate that they are incorporated into the tumor microenvironment and can stimulate tumor growth. Their biodistribution and tumor tropism, however, may also be exploited to target tumors, e.g., using a suicide transgene approach.

## Recent developments: exosomes, microparticles and MSCs

As with many other cell types, MSCs are capable of forming exosomes [63, 93, 94]. Exosomes are small membrane vesicles (40–100 nm in diameter) of endosomal origin derived from MSCs. Exosomes have been found to accumulate in target cells of MSC therapy, such as tubular cells in acute kidney injury [63], or after recovery from traumatic brain injury [95]. In other studies, microvesicles have been found to contain signaling molecules which are hypothesized to be important for MSC-mediated therapeutic effects by horizontal transfer, such as miR-133b in a rodent stroke model [96], or insulin-like growth factor receptor in renal tubular injury [97]. Kordelas et al. [98] administered exosomes isolated from MSCs to a patient with severe graft-versus-host disease; this patient showed marked improvement after the exosome infusion. This field is currently expanding rapidly, and can only be covered briefly by this review. One of the relevant open questions for the biodistribution of MSCs is whether exosomes are indeed formed by intravasally administered MSCs.

## Summary: possible ways for MSCs to interact within the local environment of the bloodstream to direct their biodistribution

**Fig. 1:**
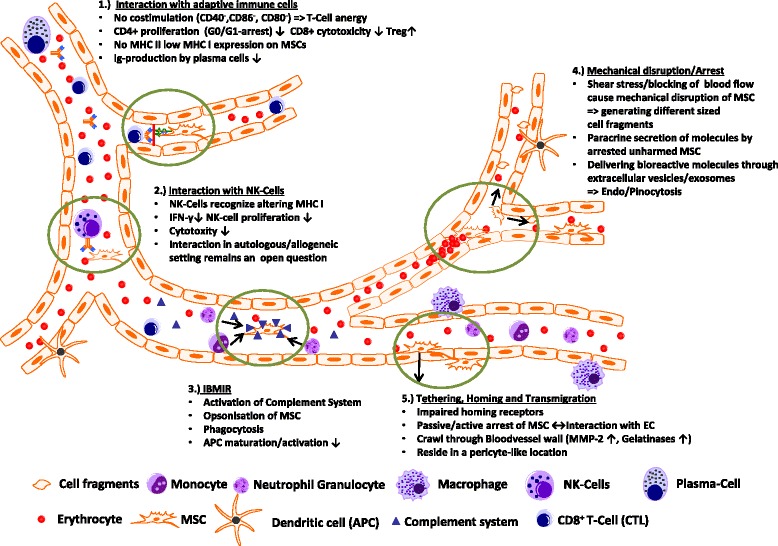
Possible ways that MSCs interact within the local environment of the bloodstream. Descriptions of cell types are shown below and the possible interactions are circled in *green. APC* antigen-presenting cell, *EC* endothelial cell, *IBMIR* instant blood mediated inflammatory response, *IFN* interferon, *MHC* major histocompatibility complex, *MSC* mesenchymal stem/stromal cell, *NK* natural killer, *Treg* regulatory T cell

A summary of the possible ways MSC might interact within the blood circulation is shown in Fig. 1. MSC surface marker profiling has revealed no expression of the co-stimulatory molecules CD40, CD86, and CD80 needed for correct T-cell responses leading to T-cell anergy. In vitro studies also showed that CD4+ T cells in contact with MSCs were arrested in the G1/G0 phase and stopped proliferating whereas regulatory T cell proliferation was favored and IgG production by plasma cells seemed to be affected [46]. In addition, MSCs only express a low amount of major histocompatibility complex (MHC) I and almost no MHC II (except after interferon-γ treatment), making them more evasive to NK cell cytoxicity in an allogenic/xenogenic setting. Interactions between NK cells and MSCs in general have been controversial, as discussed by different groups (e.g., [99–101]). MSCs seem to lower NK cell cytoxicity through downregulation of interferon-γ expression and production of anti-inflammatory IL-4 and IL 10, but NK cells were associated with the ability to lyse MSCs from allogenic donors [99]. Additionally, the so-called instant blood-mediated inflammatory response might be triggered by the innate immune response caused by tropism of dying MSCs within the blood circulation, resulting in complement activation and opsonization of injected MSCs following uptake of marked MSC cell fragments by primary/secondary phagocytes, as was shown by Moll et al. [102]. Intravital microscopy of MSCs in a cremaster muscle mouse model (our unpublished data) revealed that MSCs are likely to be disrupted by the shear force of the blood flow, resulting in fragmentation of the cell and creation of small extracellular vesicles able to influence paracrine secretion of immunomodulatory molecules or cause phagocytosis of these fragments by macrophages and endothelial cells, subsequently followed by clearance of disrupted MSCs in the liver and spleen within a few days. MSCs that find a niche and survived the journey through the bloodstream might interact actively or passively with the endothelial wall and may extravasate after interacting with the extracellular matrix (e.g., with MMP 2 and gelatinase) and reside in a pericyte-like location in the long term.

## Conclusion

The final fate of the bulk of i.v. injected MSCs remains elusive, since preclinical animal studies and some human data have been able to detect only small proportions, if any, of injected MSCs. A number of open questions remain. These include: Which contacts are made between MSCs and other cells upon infusion in the bloodstream and what are the consequences of these? What is the fate of MSCs that do not migrate into inflamed tissue and are there physiological clearance pathways for transplanted MSCs? Given that many therapeutic effects have been observed without detectable MSCs in the target tissues, are intact MSCs therefore relevant for the observed effects?

We believe that further careful analysis of animal disease models, including investigation of the role of mediators such as exosomes, signaling proteins, and microRNAs, will help further advance our understanding of why we have so far not obtained clear answers about how MSCs biodistribute, migrate and home, and how these cells exert their beneficial effects, and what might be the potential of these new insights for the development of further improvements of MSC-derived therapies.

## Note

This article is part of a thematic series ‘Mesenchymal Stem/Stromal Cells—An update’. Other articles in this series can be found at http://www.biomedcentral.com/series/mesenchymal

## Abbreviations

BM-MSC: Bone marrow-derived mesenchymal stem/stromal cell
DC: Dendritic cell
GFP: Green fluorescence protein
hMSC: Human mesenchymal stem/stromal cell
i.v.: Intravenous/intravenously
IL: Interleukin
MHC: Major histocompatibility complex
MSC: Mesenchymal stem/stromal cell
NK: Natural killer
TLR: Toll-like receptor
TNF: Tumor necrosis factor
VCAM: Vascular cell adhesion molecule
